# One hub-one process: a tool based view on regulatory network topology

**DOI:** 10.1186/1752-0509-2-25

**Published:** 2008-03-04

**Authors:** Jacob Bock Axelsen, Sebastian Bernhardsson, Kim Sneppen

**Affiliations:** 1 Centro de Astrobiología, Instituto Nacional de Técnica Aeroespacial, Ctra de Ajalvir km 4, 28850 Torrejón de Ardoz, Madrid, Spain; 2 Department of Theoretical Physics, Umeå University, 901 87 Umeå, Sweden; 3 Center for Models of Life, Niels Bohr Institute, Blegdamsvej 17 DK-2100 Copenhagen Ø, Denmark

## Abstract

**Background:**

The relationship between the regulatory design and the functionality of molecular networks is a key issue in biology. Modules and motifs have been associated to various cellular processes, thereby providing anecdotal evidence for performance based localization on molecular networks.

**Results:**

To quantify structure-function relationship we investigate similarities of proteins which are close in the regulatory network of the yeast Saccharomyces Cerevisiae. We find that the topology of the regulatory network only show weak remnants of its history of network reorganizations, but strong features of co-regulated proteins associated to similar tasks. These functional correlations decreases strongly when one consider proteins separated by more than two steps in the regulatory network. The network topology primarily reflects the processes that is orchestrated by each individual hub, whereas there is nearly no remnants of the history of protein duplications.

**Conclusion:**

Our results suggests that local topological features of regulatory networks, including broad degree distributions, emerge as an implicit result of matching a number of needed processes to a finite toolbox of proteins.

## Background

Contemporary systems biology have provided us with a large amount of data on topology of molecular networks, thereby giving us glimpses into computation and signaling in living cells. It have been found that 1) regulatory networks have broad out-degree distributions [1,2], 2) transcriptional regulatory networks contains many feed forward motifs [3], and 3) highly connected hubs are often found on the periphery of the network [4]. These findings are elements in understanding the topology of existing molecular networks as the result of an interplay between evolution and the processes they orchestrate in the cell.

In this paper we consider properties of proteins in the perspective of how they are positioned relative to each other in the network. This is in part motivated by the existence of highly connected proteins (hubs) and their relation to soft modularity [4,5] in regulatory networks. In particular one may envision broad degree distributions and possible isolation of hubs as a reflection of a local "information horizon" [6] with partial isolation between different biological processes. We here address this problem by considering the yeast regulatory network [7] with regards to protein properties. Using the Gene Ontology (GO) Consortium annotations[8] we will show that locality in the regulatory network primarily is associated to locality in biological process, and only weakly related to functional abilities of a protein.

## Results

**Figure 1:**
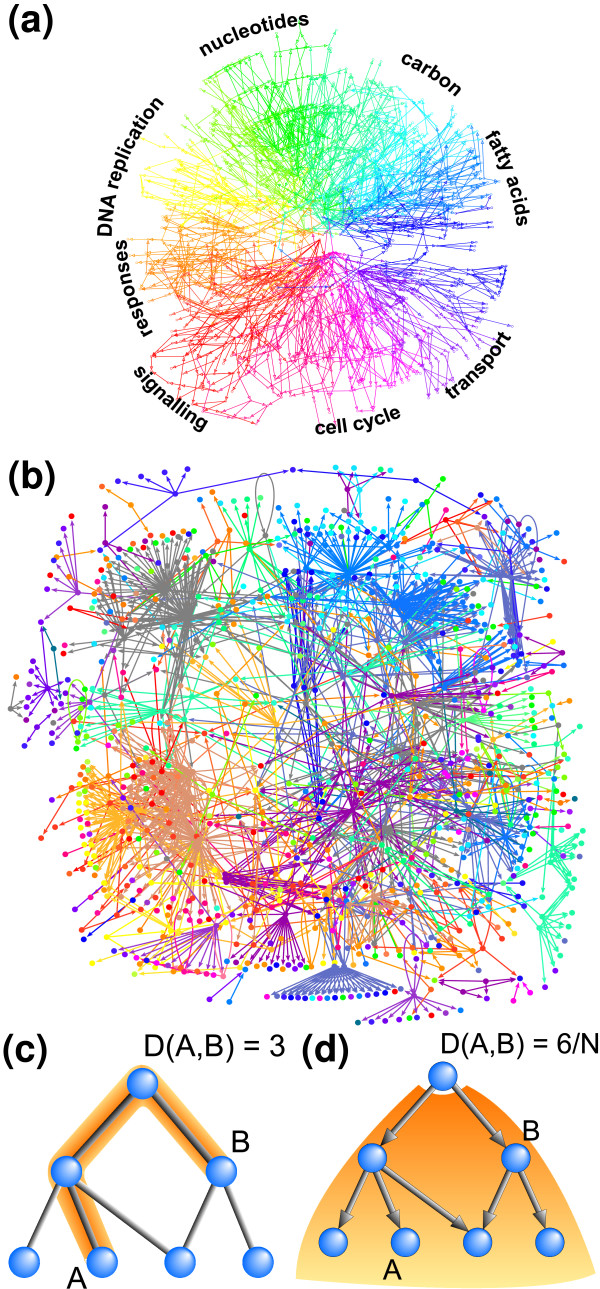
**(a) Gene Ontology (GO) annotation graph for biological processes.** The network is color coded according to overall classification of different processes. (b) Regulatory network of S. Cerevisiae, including known transcription and enzymatic interactions. The nodes represent proteins which have been colored according to their position in the above GO-graph. (c) The direct distance between two nodes A and B in a GO-annotation network is the length of the shortest path between the two nodes using the breadth-first search method, disregarding directions. Since each protein could have several GO-annotations, the distance between a pair of proteins is the shortest among all possible assigned annotations. (d) the hierarchical distance between node A and B is defined from the smallest total downstream region *n*(*A, B*) of any node that include both A and B. The hierarchical distance is the normalized *D*(*A, B*) = *n*(*A, B*)*/N *where N is the number of GO-nodes that has a protein in the shown regulatory network. D(A,B) captures that the distance between A and B is smaller if one is below the other, than if they are on separate branches.

Figure 1 show the regulatory network [7] for the yeast *Saccharomyces Cerevisiae *and the color coded GO-graph for annotations of biological processes. The GO-graph is colored such that processes that are close are colored with similar colors. The proteins in the yeast network are then colored with the color of their annotation, with hubs being colored according to the average of their targets. If the targets of a given hub take part in a very broad range of biological processes the color of the hub fades (gray). We see a fairly scattered distribution of colors, with a tendency that proteins in close proximity indeed are more similar.

More precisely, a GO-graph is an acyclic directed graph which organize proteins according to a predefined categorization. A lower ranking protein in a GO-graph share large scale properties with higher ranking proteins, but are more specialized. In the GO-database, proteins are categorized into three networks according to different annotations, ranking known gene products after respectively: *P) *biological process, *F) *functional ability/design of the protein and *C) *cellular components where the protein is physically located. For each of these *three *ways of categorization we examined *two *distinct ways to measure GO annotation difference (see box in Fig. 1).

**Figure 2:**
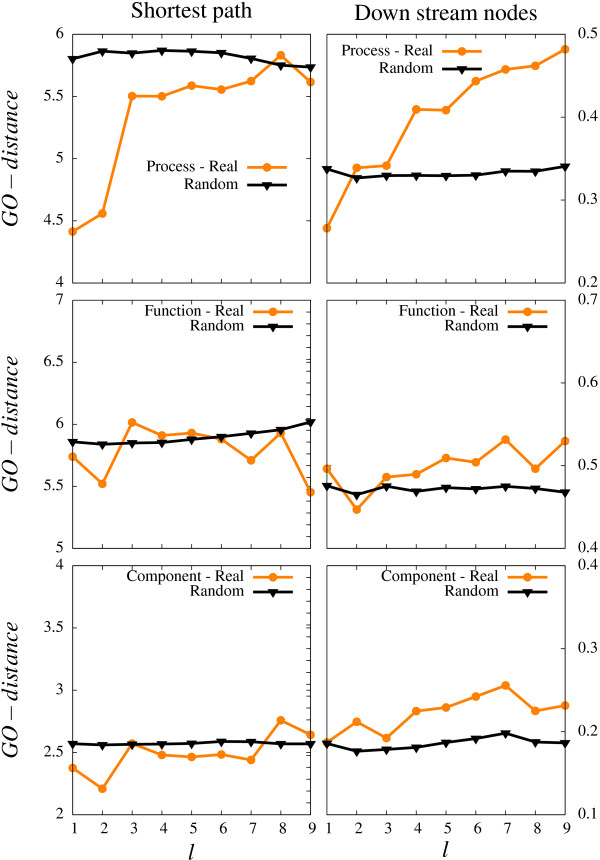
**GO-distance between two nodes as a function of separation in number of steps in the regulatory network of *S.Cerevisiae *[7].** The upper, middle and lower panel refer to respectively the *Process, Function *and *Component *GO-annotation. In left and right side of the figure we analyze respectively the direct GO-distance and the hierarchical GO-distance, as explained in Fig. 1.

Figure 2 presents the average GO-distance as function of distance *l *in the regulatory network for each of the three different GO-categories. The regulatory distance is calculated by finding the shortest path distance using breadth-first search disregarding the directionality of the links. The upper panels show that closely connected proteins are involved in closely related cellular processes, *P*. On the other hand, the middle and lower panels show a weaker relation between position in the regulatory network and *C *respectively *F *based GO-distances.

In particular Fig. 2(a) shows that proteins separated by one or two links are involved in similar processes. Here distance *l *= 1 mostly count proteins on the periphery of a hub and their directly upstream and highly connected regulator. Distance *l *= 2 count proteins regulated by the same highly connected regulator. Note that we are averaging over all pairs in the whole regulatory network including connections to less well-connected regulators. In this way the highly connected nodes are counted for each of their downstream targets and therefore the larger hubs will make the dominant contributions to this calculation.

Figure 2(b) investigate the differences in GO-annotations, but with the hierarchical distance that emphasize differences close to the root of the GO-graph for processes(P). The fact that this measure correlate to larger distances in the regulatory network implies that proteins in a larger neighborhood of the regulatory network tends to be on the same larger subbranches on the GO(P)-hierarchy.

In all the panels in Fig. 2 we also compare to a null model, generated by keeping the regulatory network, but randomly reassigning which proteins from the GO-graph that are assigned to which positions on the network. This randomization maintain the positions of all nodes in the regulatory network exactly. By doing this randomization one loose any *P, F *or *C *correlation between a regulator and its downstream targets. Any conceivable GO-distance therefore becomes independent on the regulatory distance.

**Figure 3:**
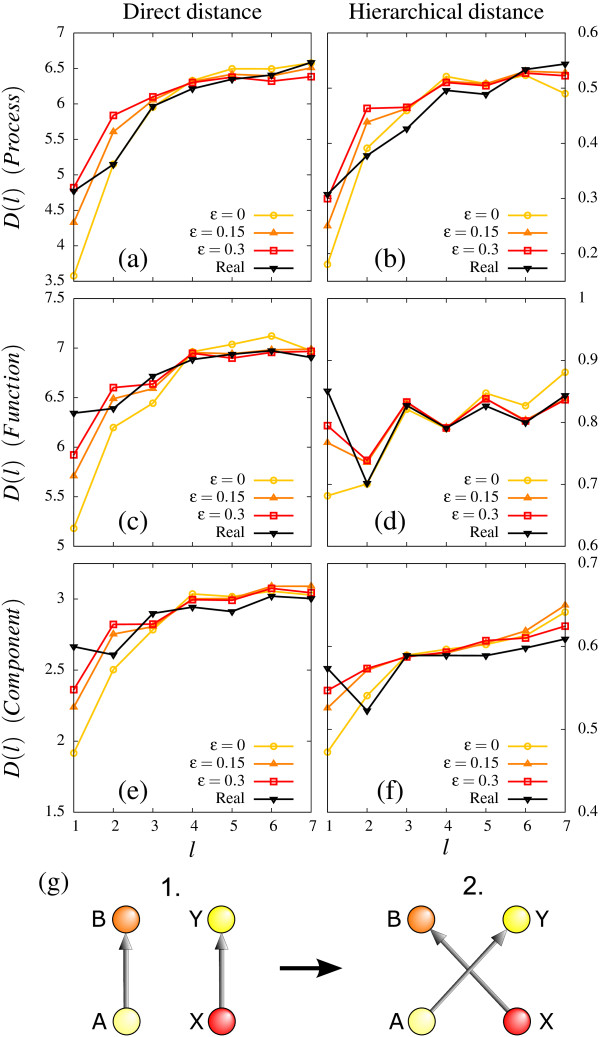
**Average GO-distances for biological process, molecular function and cellular component for the regulatory network of S. Cerevisiae (black curve), and its randomized counterparts.** As *ε *→ 0 one increase the bias for generating random networks with maximal proximity (similarity) of the GO-annotations of neighboring proteins. Left column (a,c,e) analyze the direct GO-distances, whereas the right column (b,d,f) analyze the hierarchical GO-distances as function of distance in the real and the randomized regulatory networks. In (g) is shown how we randomize the networks with probability *ε *: A random regulatory network is generated from the real one by multiple rewirings of pairs of regulatory links. For each rewiring one select two random connections A → B, X → Y and consider rewiring to a network where instead A → Y and X → B. With probability *ε *one always rewire. With probability 1 - *ε *one finds a random pair of links where the GO-distances after are smaller or equal to the GO-distances before the swap. That is, D(A,Y) ≤ D(A,B) and D(X,B) ≤ D(X,Y), here symbolized by nodes of similar colors being brought closer together.

Figure 3 quantify the correlations observed for Fig. 2(a) and 2(b) by comparing with another null model, which explicitly conserves the GO annotations but allow for complete reorganizations of the transcription network. That is, we generate families of null models by randomizing the regulatory networks while maintaining the in- and out-degree for the nodes and with a bias for neighborhood correlations of a GO annotation (see Fig. 3(g)). In detail, for a bias parameter *ε *= 0, the correlations are maximal given the available nodes in the original network. For finite *ε *there are imperfections in the sampled networks, which implies that there is some probability that the link rewiring increases the GO distance. Figure 3 show resulting GO-distances as a function of distance in the yeast network for three values of *ε*.

From Fig. 3(a) we see that in order to reproduce the observed local correlations of GO(P) in a random sample of networks, these need to be generated with maximal bias. That is, the network generated with *ε *= 0 reproduce observed correlations between processes of proteins which are downstream of the same regulator *i.e*. at distance *l *= 2 in the regulatory network. At distances *l > *2 there are no detectable correlations, which in turn is reproduced by allowing small imperfections (*ε *~ 0.15) in the rewiring.

In Fig. 3(b) we repeat the investigation from a), but with respect to the hierarchical GO(*P*) distance. In this case we see that *ε *~ 0.15 → 0.30 reproduce the observed correlations between protein processes out to larger regulatory distances (*l *~ 3). Figure 3(c)–(f), on the other hand, show that function or cellular localization are only moderately related within the same hub (*l *~ 2), and unrelated at all larger distances.

## Discussion

Protein regulatory networks are highly functional information processing systems, evolved to perform a diverse sets of tasks in a close to optimal way. It is of no surprise that they are not random, also in ways that can be detected without knowing much about what actually goes on in the living system they regulate. However we do not, a priori, know much about the relative importance of function versus history: Is the topology of a network primarily governed by the processes it direct, or is its topology influenced by random gene duplications [9,10] and "link" rewirings [11]?

Concerning gene duplications [9,12-19], we detected 581 paralogous pairs among the 848 gene products in YPD, see methods. Of these 581 pairs, only ~15% significantly retained their common regulator, and only ~0.6% of the proteins pairs at distance *l *= 2 are detectable paralogs. Therefore the contribution from duplication events to any GO-similarity within hubs can be ignored.

Our analysis in Figs. 2, 3 emphasize the strong correlations between network localization and process, in particular very strong (maximally possible) correlation between process annotation of proteins in the same hub. In addition, we see some functional similarities between proteins in the same hub, in particular when considering the hierarchical GO distances at *l *= 2 in Fig. 3(d). However we also find that the functional diversity within hubs are large in terms of the direct GO distance (*l *= 2 in Fig. 3(c)). Combined Fig. 3(c,d) therefore show that proteins in the same hub have quite large direct function-GO distances, but rarely belong to entirely different function-GO categories.

In any case we emphasize that we primarily find GO-processes localized on hubs, and only weak correlations of the functional abilities between proteins involved in the same process.

The idea that process similarity are associated to network localization is not new, and implicitly behind attempts to infer gene networks from similarity in gene expression [20]. In the supplement we use gene expression from micro-arrays to re-investigate the correlation between process and locality in the regulatory network. Thereby, we provide a broader support for our findings, and present a quantitative illustration of the extent to which gene-expression studies can be used to deduce co-regulation.

Support for the ubiquity of the "one hub-one process" association is also found from the fact that the likelihood that a regulatory protein is essential is nearly independent on how many proteins it regulate [2]. That is, the question of whether a null mutant of a certain protein is viable is keyed to the essentiality of the regulated process, and not to whether the process needs many or few different "tools" to be performed.

## Conclusion

Overall we suggest that the topology of the yeast regulatory network is governed by processes located on hubs, each consisting of a number of tools in the form of proteins with quite different functional abilities. This is consistent with a network evolution where gene duplication occur, but where rewiring of regulatory links plays a bigger role [14,19,21-23]. The regulatory network is designed to co-regulate processes, and its evolutionary history must include a bias towards hub-regulation of individual processes. Degree distributions are not broad because of duplication events, but because a given biological task sometimes needs many, but typically require few tools.

Finally our analysis have consequences for development of null models for network topologies, and thereby for identifying functionally important network motifs [3]. While the previous null model [4] maintain in- and out- degrees of each protein, it ignore correlations associated to cellular process. When nearby proteins are associated to the same processes one statistically expect an increased probability for cliques [24,25]. We therefore expect that some of the many feed-forward loops in transcription networks [3] will be explained by a new type of null model: A null model where proteins contributing to a given process are forced to remain close in the randomized network.

## Methods

The GO-annotations are used without any filtering. This does not preclude bias introduced from using inferred annotations. Of the 848 genes in the YPD, 52 are not annotated and were thus not included in the analysis. 142 genes has more than one molecular function, 314 genes takes part in more than one cellular component and 463 genes participates in more than one biological process. To accommodate this the analysis was carried out by choosing the annotations which minimized the mutual distance for each pair of proteins. This choice maximally resolves significant signals, since we minimize the effect of the finite size of the GO-tree, and in the case of no signal this choice introduces no bias.

Of the 848 gene products in YPD, we found 581 paralogous pairs using BLASTP with E-value cutoff of 10^-10 ^[14,26]. For the YPD network 132 of these paralogous pairs are at distance *l *= 2. This should be compared to a null expectation of 50 ± 6 paralogous pairs at *l *= 2 found by randomizing the YPD network while keeping in- and out-degrees [4]. Therefore at max 132-50 = 82 of the paralogous pairs are in the same hub due to their history of common origin. This correspond to 82/581 ~15% of duplicated proteins in YPD. The excess of 82 paralogous pairs at distance 2 should also be compared to the total of 13554 protein pairs that the YPD network have at distance *l *= 2. Thus only ~0.6% of all proteins pairs at *l *= 2 are detectable paralogs.

As seen in our Additional file 1, we reach the same basic conclusion of hubs being functionally isolated using a completely different approach based on gene expression data. Analyzing micro-array data from 482 stress experiments from Saccharomyces Genome Database [27] and managing the false discovery rate as in [28] we indeed find localization of perturbations on our regulatory network. Thus the appendix supports the robustness of our results to an independent categorization of protein processes.

## Authors' contributions

All authors contributed equivally to this work. All authors read and approved the final manuscript.

## Supplementary Material

Additional file 1 Correlating microarray data of stress conditions with the YPD. Using 465 microarrays of stress conditions for S. Cerevisiae, from Stanford Genome Database, we perform a statistical analysis showing that functions are localized in the regulatory network. Click here for file
