# Supplementary material for: One hub-one process: a tool based view on regulatory network topology
Source: BMC Syst Biol. 2008 Mar 4;2:25. doi: 10.1186/1752-0509-2-25 (PMC2292138; doi:10.1186/1752-0509-2-25)
Supplement: Additional file 1 — Correlating microarray data of stress conditions with the YPD. Using 465 microarrays of stress conditions for S. Cerevisiae, from Stanford Genome Database, we perform a statistical analysis showing that functions are localized in the regulatory network. [file 1752-0509-2-25-S1.pdf]

I. SUPPLEMENTARY MATERIAL

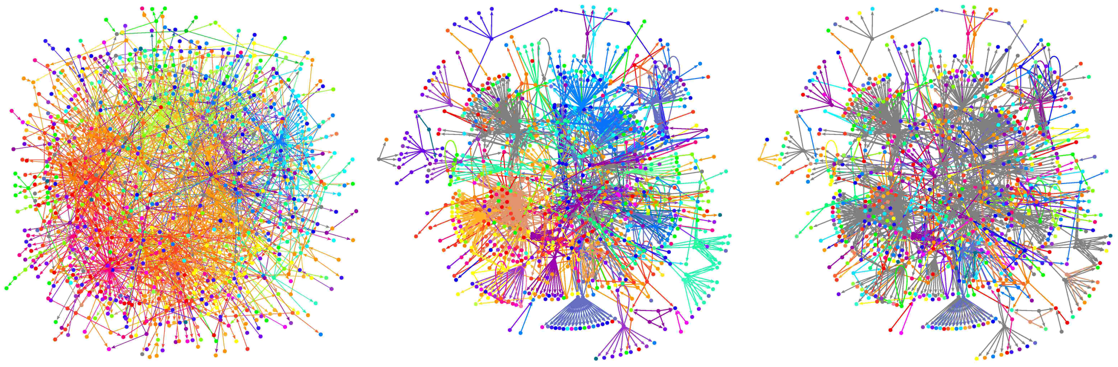

FIG. 1: (a) Rewiring the YPD network by bringing nodes of similar biological process annotation closer together. (b) The real YPD network. (c) The YPD network with randomized GO annotation for biological processes.

In Fig. 1 we have shown alternative ways of communicating the findings in the paper. In Fig. 1(b) we display the YPD network from Fig.1 in the main paper. To visualize that the GO annotations are indeed optimized and non-random we have in Fig. 1(c) shown the YPD network where we have reshuffled the GO annotations. Since the hubs are annotated to a collection of randomly selected biological processes they will all appear as overall ambiguous (gray) while all enzymes (end nodes) appears perfectly mixed in a harlequin-like fashion. In contrast, the clearly separated functional neighborhoods in the real network in Fig. 1(b) leaves most of the hubs with a easily identifiable color. Conversely, it is natural to ask if the YPD network is optimized according to GO-annotation proximity. In Fig. 1 we have rewired the YPD network in order to bring nodes that share biological processes closer together. Compared to the real network the effect is a clearer functional separation, but there is a trade-off in the resemblance of the topology.

Ref. [1] and [2] investigated the hierarchical properties of networks with broad-degree distributions. The hierarchy index $\mathcal{F}$ was defined as the fraction of hierarchical paths out of the total number of paths. A hierarchical path is a path where the hierarchy is preserved along the path in the sense that low-ranking nodes always receive orders and never gives orders.

Using the hierarchy index, $\mathcal{F}$, we can clarify the trade-off in Fig. 1(a) by calculating the $\mathcal{F}$ value disregarding directionality and finding it to be $\mathcal{F} = .49(3)$. The real YPD network in Fig. 1(b) has $\mathcal{F} = .26$. The network in Fig. 1(c) is per definition topologically identical to the

real network in Fig. 1(b). For an ensemble of randomly rewired versions of the YPD network we found $\mathcal{F} = .60(3)$. Thus, when optimizing the YPD network according to biological process neighborhoods we partly loose the observed hub-hub separation. Therefore we conclude that the model is extremely simple and is not able to capture both functional neighborhood optimization and soft modularity.

We also investigated the relationship between locality and function by the use of microarray results from yeast cultures subjected to different stress conditions. This choice was motivated by the fact that expression of enzymes during stress shows the strongest response in terms of variance and expression fold. In contrast, the variance and fold change of regulators regulating other regulators has low signal-to-noise using microarrays.

482 microarray stress experiments on *Saccharomyces Cerevisiae* were downloaded from Stanford Genome Database [3]. These experiments covered the following conditions: heat shock, osmotic shock, diauxic shift, hydrogen peroxide shock, Menadione, diamide, sorbitol, DTT, amino acid starvation, MMS, Nitrogen depletion, Na+, Phosphate, Sulfate, Uracil, sugar enriched media, gamma irradiation, YPD medium inoculation and others. The data was centered for each gene.

We hypothesized that the correlation between expression of a given protein and its network neighborhood would show the strongest average signal if we focused on the hubs and their targets. That is, the large number of target proteins of hub-regulators will enhance any functional locality in the signal compared to the bulk signal at larger distances.

We compared the overlap of all pairs of hubs larger than size 10 and then removed the hubs that had a larger target overlap than 1/3 of the targets of another, larger hub. This ensured that functional response overlap would not be from network overlap. In this way we ended up with a total list of 26 isolated hub regulators from the regulatory network of yeast.

We detected responsiveness of a hub by a combination of statistical tests and biological reasoning. The target set of enzymes of a hub was considered as a supervised cluster to be tested against the bulk of the network. The variance of each experiment in the cluster versus the rest of the network was analyzed by using a t-test. To manage the false discovery rate for the multiple comparisons we use the method of [4] with a conservative p-value threshold of 0.5%. If any of the resulting, significantly upregulated experiments showed at least a two-fold upregulated response as well, the hub was dubbed responsive to stress. In this fashion only 12 out of the 26 selected hubs were found responsive. Figure 2 shows the total average response resolved by distance. In this figure the cluster is responsible for amino acid synthesis according to the available biological

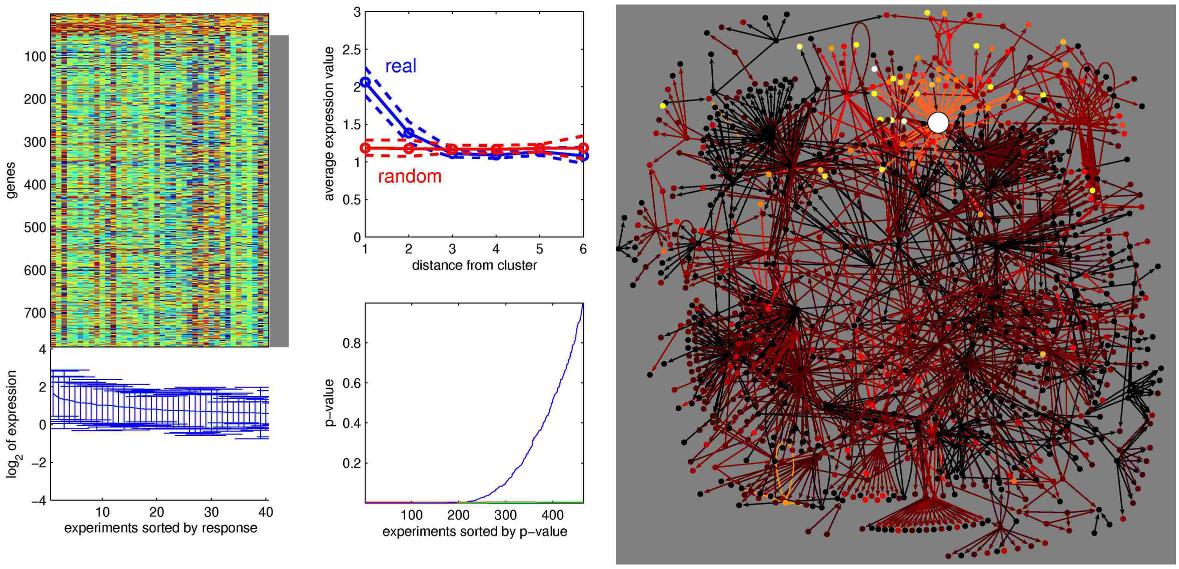

FIG. 2: The responsiveness filter (here shown for the weakest responding cluster): sort experiments according to response in selected cluster and perform *t*-test with 0.5% p-value cut-off, correct for false discoveries and finally only accept two-fold responses. Left: the data for the cluster (top of matrix) and the rest of the genes (bottom/gray sidebar). The color scheme is red for expression two-fold enhanced, green for zero change and blue for two-fold depressed. The curve and error bars are the average experimental values in the cluster sorted in descending order from right to left (in $\log_2$ transformed format). Middle: the average response resolved by distance from the hub in question, here the hub is GCN4. The blue curve and dashed errors is the real signal, and the red curve is a random expectation created by randomly swapping the expression data for the genes. There is a clear signal for the targets of the hub and a weak signal for two-steps away. Mid-low is the false discovery management procedure as referred to in the text. Right: the response mapped onto the Yeast Regulatory network with a "hot" color scheme where light yellow is strong response and dark red is no response. The tested cluster shows a clear locality resolved response.

process GO-annotation. Our analysis finds that the experiments that most strongly activates this cluster are amino acid starvation, and nitrogen depletion type experiments. This supports the overall postulation of the paper that network locality means functional locality and thus points to local hierarchies of hubs and their targets as natural "soft" modules in biological regulation.

For the responsive hubs the experiments were sorted in descending order according to the average upregulation in the cluster. For the 20 first experiments in each list the average response per distance was calculated. Notice that we here include experiments irrespectively of the outcome

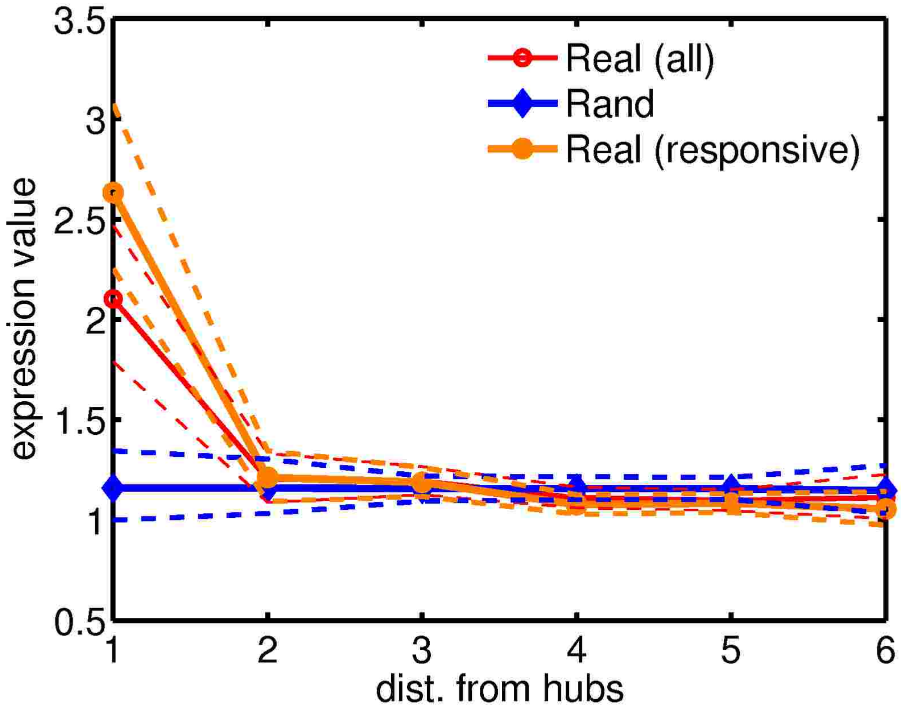

FIG. 3: Total average response to stress in the yeast regulatory network. The blue diamonds is the random expectation created by random swapping the expression data for the genes. The red circles is the average response for all 26 clusters for the 20 first experiments in the sorted list. The orange dots is the average response for the 12 clusters that were found to be responsive according to our criterion(see text). As can be seen the signal is clear for local clusters and then fades rapidly further away.

of the responsiveness filter. This is to avoid selecting experiments that *only* activates the cluster and nothing else, a reasonable choice since we are interested in functional locality of a neighborhood at different distances. In Fig. 3 we show the total average response of the stress responsive hubs resolved by distance and compared to random expectation. In this figure we see a clear local functionality on average, which naturally does not account for co-activation effects.

In Fig. 4 we show the stress response mapped onto the network for each cluster. The co-activation of clusters is obvious. The annotation of the biological processes also raises the expectation of such co-activation. For example, HAP2, GAL4 and MIG1 are all related to the utilization of carbon sources and would be expected to co-activate to a certain degree. This is somewhat visible in the graphs as an overall activation of the same regions for those three hubs.

A more tangible analysis of the modular co-activation is shown in Fig. 5. Here the raw data is

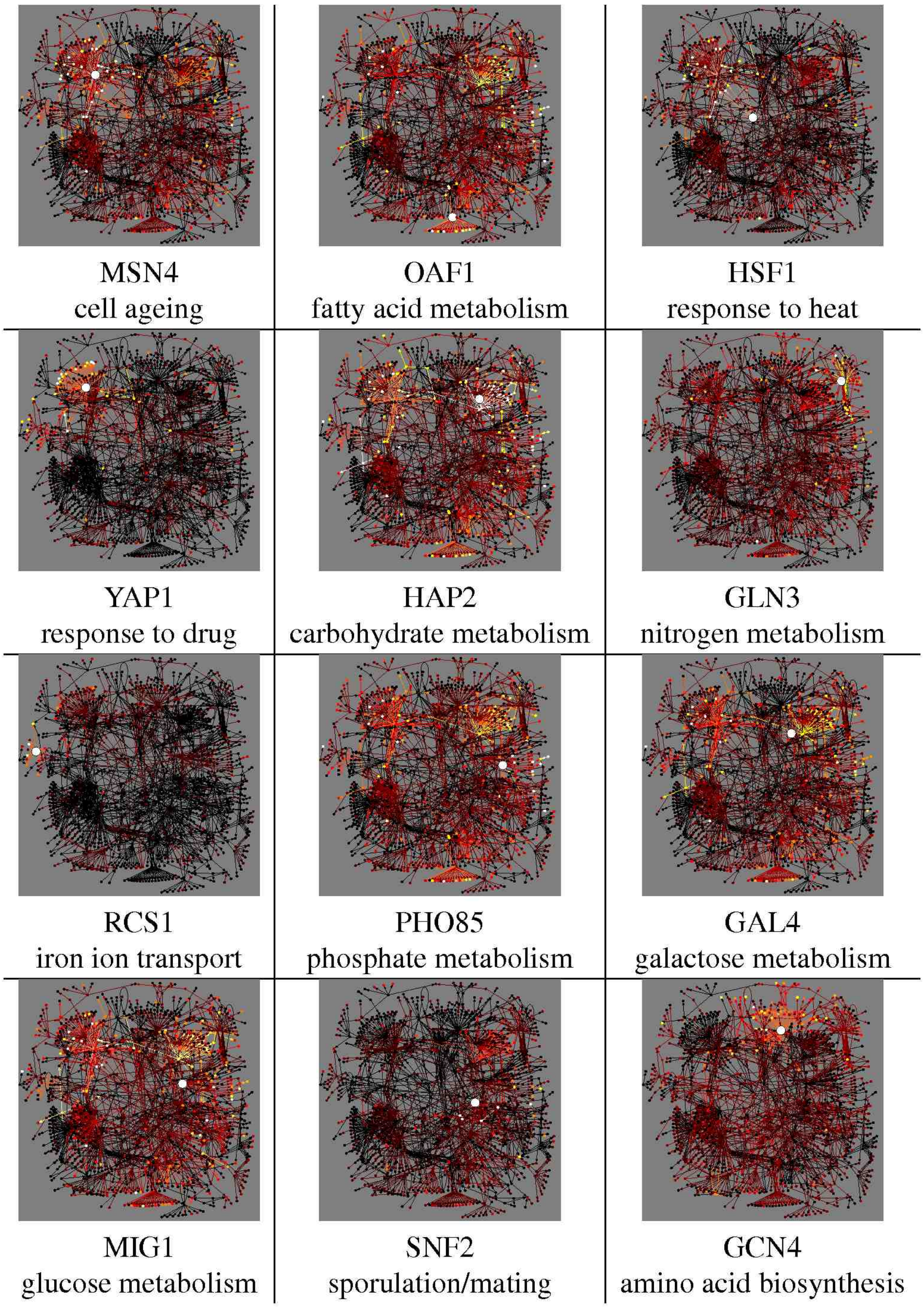

FIG. 4: The 12 most responsive hubs and clusters. The organization is: strongest response in the upper left corner and then descending to lower right corner. The name of the hub and the biological process annotation is indicated below each graph. The color scheme is ”hot” meaning strongest response is white and weakest response is dark red. The hub of the investigated cluster is enhanced and colored white for each graph. The locality of the signal is clear, but there is also often a clear co-activation. Further, as can be seen there is a region, located around the middle and lower left of the network which is almost always silent in stress conditions.

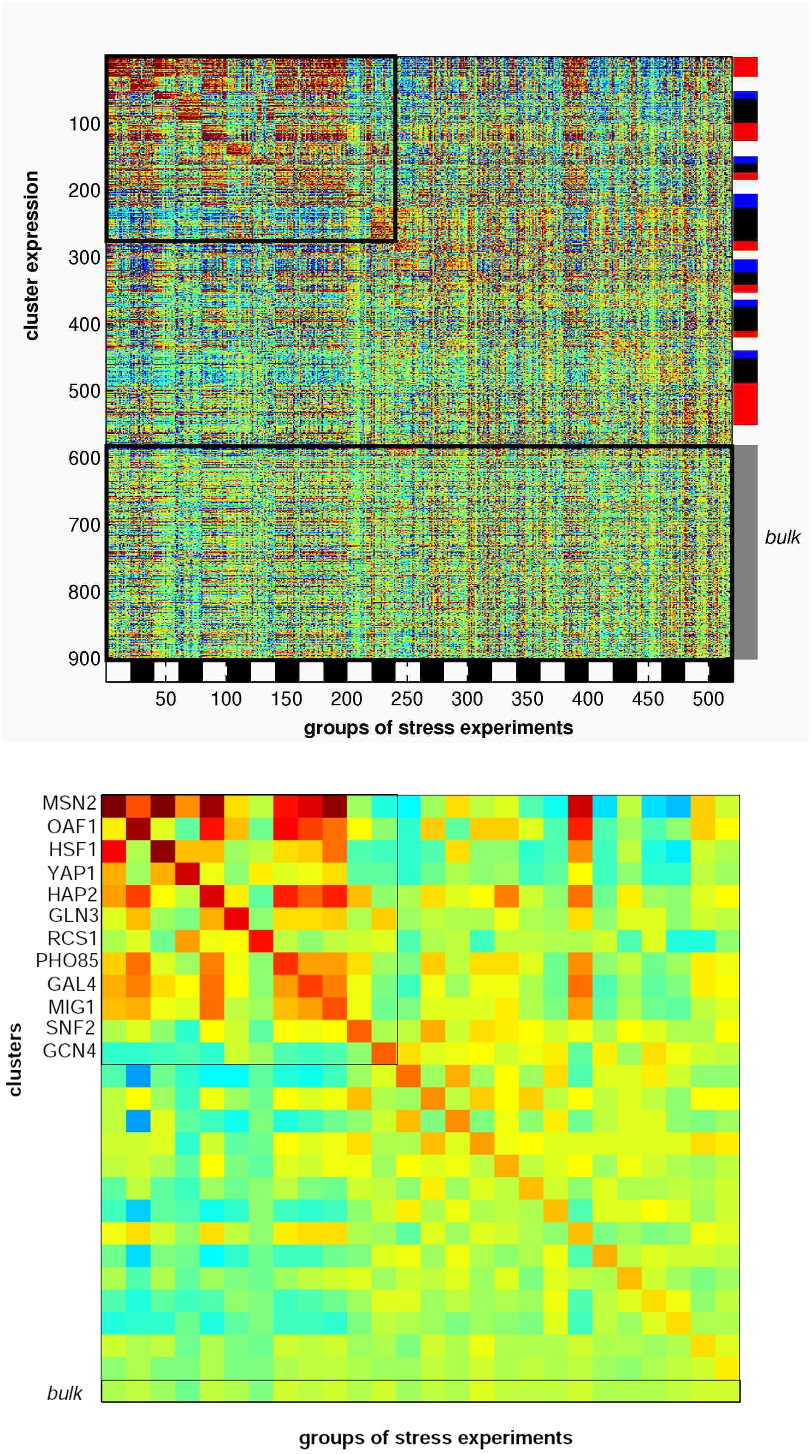

FIG. 5: Investigating co-activation of clusters sorted by response strength descending from upper left corner. Top: the raw data for each of the 26 clusters. The color bar to the right delineates the groups of genes belonging to each cluster. The black and white color bar at the bottom demarcates the groups of the 20 strongest activating experiments for each cluster. Bottom: the raw data has been coarse grained by taking the average in each bin. The name of the cluster has been indicated to the right. For both matrices the responsive clusters are indicated with the black box at the upper left corner. The black box at the bottom is the response of the genes not in any cluster. The color scheme is red for strong activation (larger than 2), blue for deactivation (less than .5) and green for no activation.

shown in an expression matrix with rows indicating genes and columns indicating experimental conditions. The second matrix is a coarse-grained version of the raw data for ease of analysis. Next to the raw data matrix is a color bar indicating the number of genes in each cluster including the non-responsive ones. A black box in the upper left corner separates the responsive clusters from the rest. Focusing on the coarse grained lower matrix the picture becomes clear: there is a large group of of more or less co-activated clusters with MSN2, OAF1, HSF1, HAP2, PHO85, GAL4 and MIG1 as central members, YAP1 and GLN3 as semi-correlated members and three clearly independent clusters: RCS1, SNF2 and GCN4. Since we have removed network overlap in the initial choice of hubs and clusters the co-activation stems from a combined reaction to the most stressful conditions. As expected above the clusters HAP2, GAL4 and MIG1 are indeed seen to be co-activated in this analysis. The co-activation clusters are often separated in the network, thus underpinning the point about the modules serving as tools for the organism to be employed according to need.

Finally we investigating the full biological process GO-annotation for the 12 clusters along with the SGD experiment access codes and description.It becomes clear that the large co-activated cluster arises from heat shock experiments that triggers protein folding responses (HSF1), cell aging (MSN2), membrane reconstitution (OAF1) and energy production (HAP2, PHO85, GAL4 and MIG1). This is a coordinated response resulting from external stimuli triggering many independently regulated needs. Furthermore, the semi-independence of YAP1 is the specialized tools that the regulator controls and thus it is mostly triggered by hydrogen peroxide and diamide shocks. The semi-independence of GLN3 comes mostly by starvation of nitrogen, amino acids and adenine. The purely independent response of RCS is ambiguous, since none of the stress conditions in our database matches the iron ion function of this cluster. SNF2 is seemingly activated by phosphate depletion which triggers a mating-type phenotypic switch. And finally, GCN4 is clearly activated by a pure setup of amino acid and nitrogen starvation conditions creating a demand for biosynthesis of these compounds.

[1] J. B. Axelsen, S. Bernhardsson, M. Rosvall, K. Sneppen, A. Trusina, *Phys. Rev. E. Stat. Nonlin. Soft. Matter. Phys.* **74**, 036119 (2006).

[2] A. Trusina, S. Maslov, P. Minnhagen, K. Sneppen, *Phys. Rev. Lett.* **92**, 178702 (2004).

[3] SGD project. "Saccharomyces Genome Database" `ftp://ftp.yeastgenome.org/yeast/`

[4] Y. Benjamini, D. Drai, G. Elmer, N. Kafkafi, I. Golani, *Behav. Brain. Res.* **125**, 279 (2001).
